# ECPR combined with CRRT successfully rescues a patient who experienced sudden cardiac arrest for 152 minutes: A case report

**DOI:** 10.1097/MD.0000000000041298

**Published:** 2025-01-31

**Authors:** Ya-Dong Wang, Jin-Feng Lin, Zhi-Long Cao, Su-Yan Zhang, Xu-Dong Han

**Affiliations:** a Department of Critical Care Medicine, Nantong Third People’s Hospital, Nantong, China.

**Keywords:** brain resuscitation, cardiac arrest, cardiology, cardiopulmonary resuscitation, case report, CRRT, ECPR, ventricular fibrillation

## Abstract

**Rationale:**

Cardiac arrest (CA) is a life-threatening event with a high mortality rate, and neurological injury following cardiopulmonary resuscitation (CPR) is a leading cause of death and disability in survivors. While prolonged CPR is often associated with poor neurological outcomes, there is limited evidence of successful recovery following extended resuscitation efforts. This study aims to highlight the potential for recovery after prolonged CPR by reporting a case of a patient who underwent 152 minutes of CPR, regained consciousness, and made a full recovery. The purpose is to explore whether advanced life-support techniques, such as extracorporeal CPR (ECPR), can improve survival and neurological outcomes even after prolonged CA.

**Patient concerns:**

A 53-year-old man with no prior health issues experienced sudden CA while exercising and underwent prolonged CPR.

**Diagnoses:**

Restoration of spontaneous circulation following CA and ventricular fibrillation.

**Interventions:**

ECPR, target temperature management, continuous renal replacement therapy, and intracranial pressure management.

**Outcomes:**

Immediate recovery: following the restoration of spontaneous circulation, the patient was immediately transferred to the intensive care unit for further treatment. Despite the prolonged CPR duration, the patient remained hemodynamically stable and was able to tolerate the intensive interventions. Neurological recovery: after 1 week of intensive therapy, the patient regained consciousness. Initially, there was some confusion and disorientation, but he gradually became fully alert, oriented, and communicative. Neurological assessments indicated no significant long-term deficits, and brain imaging showed no signs of irreversible damage. Cardiological and renal recovery: cardiac function was closely monitored, with no evidence of significant ischemic damage to the myocardium. The patient’s renal function improved with continuous renal replacement therapy, and kidney function returned to normal following the discontinuation of dialysis. Discharge: after 2 weeks of treatment in the intensive care unit and a transfer to the cardiology department for rehabilitation, the patient was discharged from the hospital. He had fully recovered both neurologically and physiologically, with no residual deficits.

**Lessons:**

This case demonstrates that prolonged CPR, when combined with advanced interventions such as ECPR, can result in favorable outcomes, including survival and neurological recovery. The findings suggest that with timely and appropriate treatment, even patients with extended resuscitation efforts may achieve full recovery, thus underscoring the potential of ECPR as a critical life-saving intervention in cases of prolonged CA.

## 1. Introduction

Cardiac arrest (CA) is a prevailing cause of death globally, accounting for 50% of all cardiovascular deaths worldwide,^[1]^ and poses a significant burden on public health. Cardiopulmonary resuscitation (CPR) is the most effective intervention to save patients experiencing CA; however, its overall success rate is only 9.9%.^[2]^ In China, the survival rate of patients with out-of-hospital cardiac arrest is only 1.5%, with even fewer patients showing good neurological outcomes upon discharge.^[3,4]^ During CA, body tissues and organs suffer severe ischemia and hypoxia, releasing inflammatory factors and various metabolic products. Upon return of spontaneous circulation (ROSC), reperfusion injury causes multiple organ dysfunction or failure,^[5]^ known as postcardiac arrest syndrome. These patients exhibit severe neurological damage and an increased mortality rate.^[6,7]^ This study aims to assess a patient who experienced CA, underwent 152 minutes of CPR, regained consciousness, and fully recovered, and he was discharged from the hospital.

## 2. Case presentation

**Figure 1.**
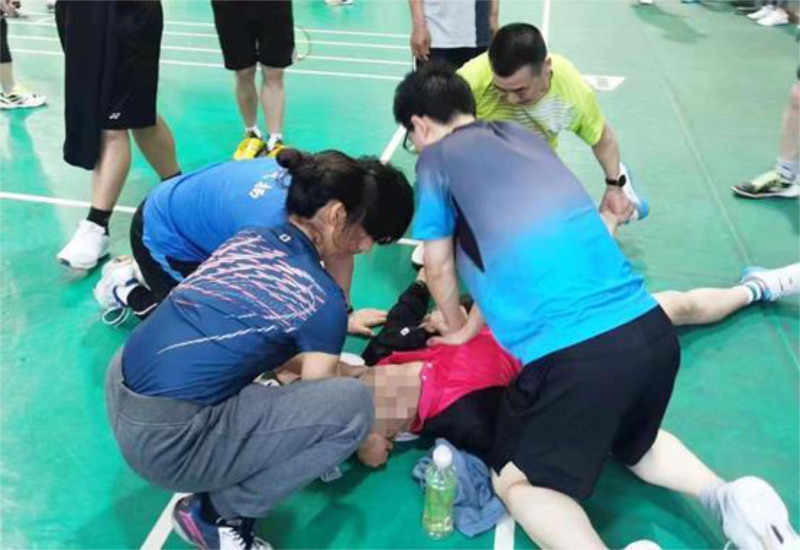
When the patient experienced sudden cardiac arrest, his friends started chest compressions.

**Figure 2.**
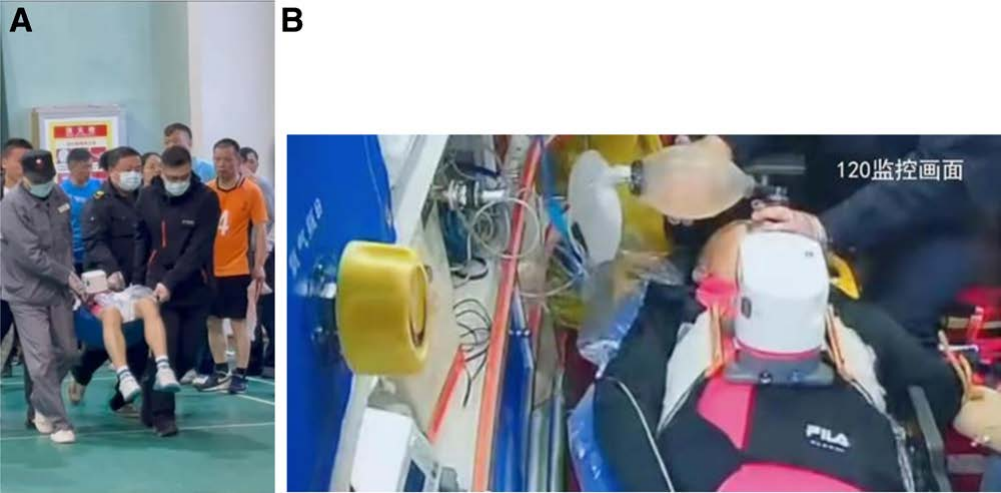
(A) Mechanical chest compression during transportation. (B) Mechanical chest compression during transportation.

**Figure 3.**
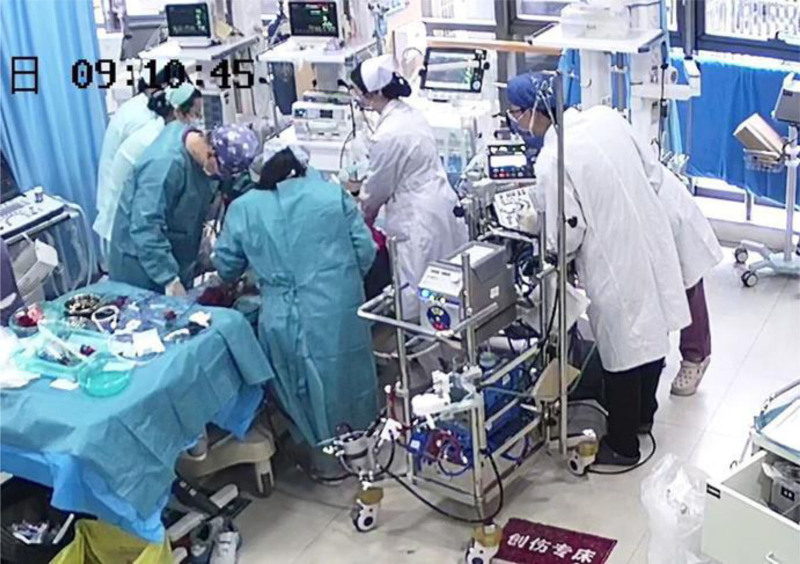
Extracorporeal cardiopulmonary resuscitation in the emergency department.

**Figure 4.**
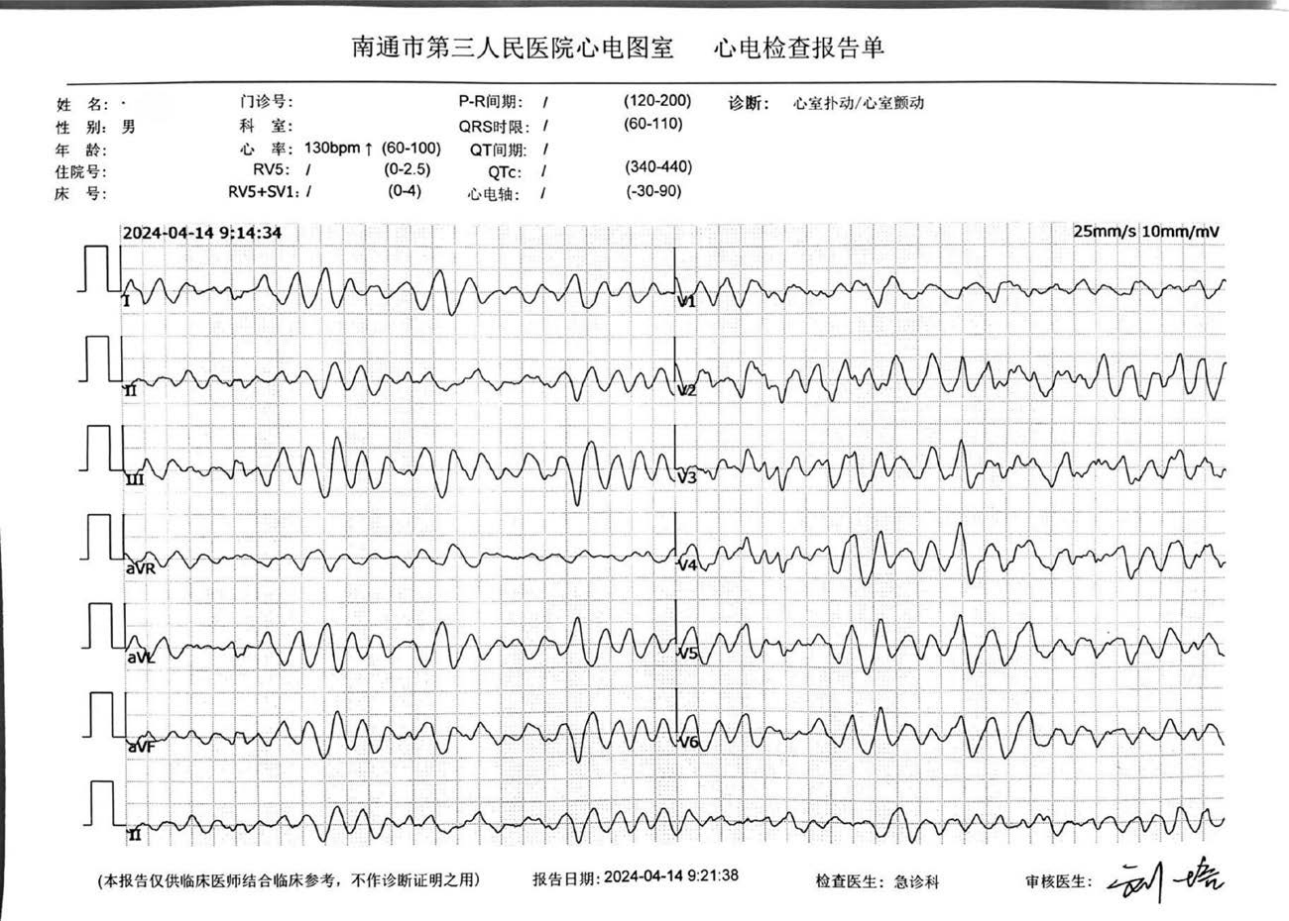
The ECG during the extracorporeal cardiopulmonary resuscitation process indicates ventricular fibrillation.

**Figure 5.**
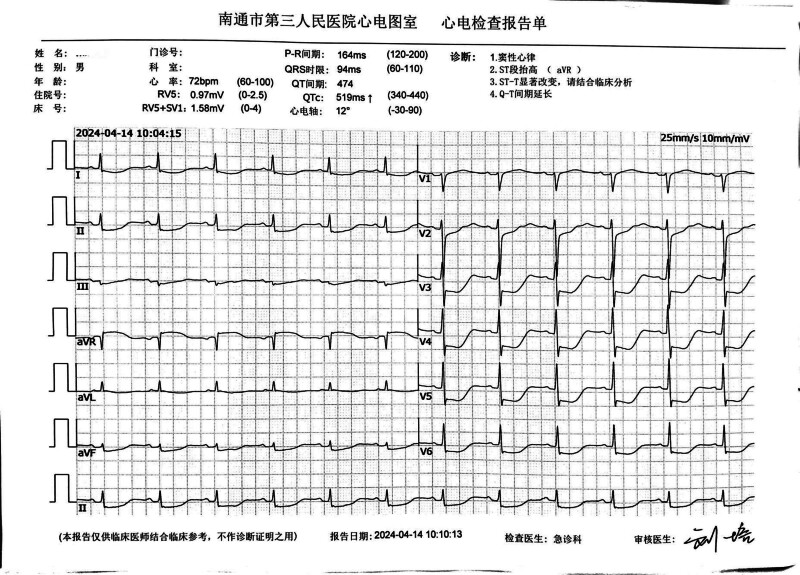
The ECG after extracorporeal cardiopulmonary resuscitation had converted to sinus rhythm.

The patient, a 53-year-old healthy male, was admitted to the Emergency Department of Nantong Third People’s Hospital on April 14, 2024. The patient experienced a sudden loss of consciousness, urinary incontinence, and dilated pupils after exercising at 07:20 that same day. His friends immediately began chest compressions (Fig. 1). The patient’s ECG was still a flat line when the ambulance arrived and emergency doctors continued compressions using the LUCAS device without interruption upon arrival and during transportation (Fig. 2A and 2B). Before arriving at the hospital, the patient did not receive defibrillation but was given 1mg intravenous doses of epinephrine twice. The patient arrived at the emergency department of the hospital at ≈07:55 am. At that point, the patient was unconscious and did not breathe spontaneously; cardiac monitoring revealed ventricular fibrillation. Two rounds of 200-J biphasic wave defibrillation and intravenous adrenaline were administered, and he was intubated for mechanical ventilation; however, cardiac rhythm was not restored. It remained a ventricular fibrillation rhythm. Consequently, venoarterial extracorporeal membrane oxygenation (ECMO) (Fig. 3) was initiated. ECMO was initiated at 08:55, with a centrifugal pump speed of 3025 rpm, an oxygen concentration of 100%, an airflow rate of 3 L/min, and a blood flow rate of ≈1.73 L/min. Owing to the ventricular fibrillation rhythm of the patient (Fig. 4), multiple 200-J biphasic defibrillation shocks were administered. Autonomous cardiac rhythm was restored at 09:52, and the color of the lips and nails gradually turned ruddy. Electrocardiography revealed sinus rhythm, elevated ST segments (aVR), significant ST-T changes, and prolonged Q-T intervals (Fig. 5), and the troponin I was 50 ng/mL. Blood pressure was ≈80/50 mm Hg while receiving norepinephrine at 30-µg/min norepinephrine. However, spontaneous breathing was absent; therefore, mechanical ventilation was continued. Subsequent coronary angiography revealed 70% stenosis in the proximal right coronary artery, no stenosis in the main left coronary artery, 90% stenosis in the distal circumflex artery, and 40% to 50% stenosis in the proximal left anterior descending artery, with a thrombolysis in myocardial infarction flow grade of 3. Therefore, no coronary artery stent was placed. The CA of the patient may have been triggered by excessive sympathetic stimulation and coronary spasms following intense exercise. After the procedure, the patient was transferred to the intensive care unit (ICU) for further treatment. Upon ICU admission, the patient was in a moderate coma, with a heart rate of 58 beats/min and arterial blood pressure of 146/98 mm Hg (with 8 µg/min of norepinephrine). Blood gas analysis revealed a pH of 7.00, PCO_2_ of 75.60 mm Hg, PO_2_ of 58.00 mm Hg, HCO_3_- of 13.40 mmol/L, base excess of −14.20, and anion gap of 32.40 mmol/L, indicating a combination of metabolic and respiratory acidosis. His lactate was 9.0mmol/L. The mechanical ventilation settings included synchronized intermittent mandatory ventilation, FiO2 50%, a respiratory rate of 12 breaths per minute, pressure control at 12-cm H_2_O, and positive end-expiratory pressure at 8-cm H_2_O, with a monitored tidal volume of ≈425 mL. Unfortunately, EtCO2 was not monitored. The patient also received continuous venoarterial ECMO treatment, active brain protection through sedation and analgesia with midazolam 5 mg/h + remifentanil 1 µg/min, a hibernation mixture (chlorpromazine 5 mg/h + promethazine 5 mg/h) combined with an ice blanket and ECMO heater to maintain a body temperature at ≈35 °C within 1 hour after send to ICU, and negative fluid balance of 100-300 mL/d to manage intracranial pressure (ICP). Owing to the acute kidney injury (urea, 8.00 mmol/L and creatinine, 146.0 µmol/L with oliguria) and metabolic acidosis of the patient, oXiris continuous renal replacement therapy (CRRT) was performed to remove inflammatory mediators and stabilize the internal environment at 13:30. Bedside echocardiography revealed a left ventricular ejection fraction of 24.44% and cardiac output (CO) of 1.442 L/min, indicating decreased CO and an optic nerve sheath diameter of 0.33 cm. The right middle cerebral artery showed peak systolic and end-diastolic velocities of 67.83 and 40.32 cm/s, respectively, a resistive index of 0.41, and a pulsatility index of 0.56, indicating no significant intracranial hypertension, but reduced cerebral blood flow. Following treatment, the patient’s hemodynamics gradually stabilized. By April 16, norepinephrine was discontinued, the left ventricular ejection fraction improved to 40.44%, and the CO increased to 2.77 L/min. The optic nerve sheath diameter was 0.34 cm, the peak systolic was 91.40 cm/s, the end diastolic was 39.52 cm/s, the resistive index was 0.57, and the pulsatility index was 1.01, indicating that no increase exists in ICP or cerebral blood flow. The ECMO tube was removed on April 18. After 2 days of oXiris-CRRT treatment, the internal balance stabilized, the interleukin (IL)-6 levels fluctuated between 12.4 and 18.5 pg/mL, and the neuron-specific enolase concentration decreased from 65.55 ng/mL (24 hours after CPR) to 61.16 ng/mL (48 hours), 28.54 ng/mL (72 hours), and 13.5 ng/mL (96 hours). During the treatment, there was no hemolysis or mesenteric ischemia. On April 19, the subhypothermia treatment was discontinued, the temperature gradually returned to ≈37 °C, and the consciousness of the patient improved, shifting from coma to drowsiness. By April 20, the tracheal tube was removed, and high-flow oxygen therapy was administered. By April 21, the patient became conscious and exhibited appropriate responses, and the limbs could move upon request. Breathing, oxygenation, and blood pressure levels were stable. By April 22, 2024, the patient was transferred from the ICU to the cardiology department. On April 29, the patient was discharged with full recovery and no neurological deficit.

## 3. Discussion

Cerebral injury following CPR is a leading cause of death and disability. Research shows that 70% of patients who achieve ROSC after CA die because of severe neurological damage.^[8]^ Therefore, minimizing postresuscitation brain injury is the main goal of treating patients with ROSC and remains a challenge in the field of CPR. In this case, the patient underwent prolonged CPR, with cerebral resuscitation efforts starting concurrently. Following the recovery of cardiopulmonary function, a significant improvement in consciousness was observed. This improvement is attributed to timely prehospital resuscitation, application of ECMO, proactive cerebral resuscitation, and management of fluid volume and the internal environment.

High-quality chest compressions are a vital link in the chain of survival during CA.^[9]^ The brain can tolerate ischemia and hypoxia for ≈6 minutes, beyond which irreversible damage occurs. In this case, medical personnel were present when CA occurred, and chest compressions were immediately initiated until emergency doctors arrived. Emergency doctors used the LUCAS device to provide continuous compression. This mechanical chest compression device can replace rescuers to provide consistent, high-quality compressions without interruption, even during transport. Consistent compressions of this quality are associated with better outcomes.^[10]^ Despite multiple defibrillation attempts, the patient remained in ventricular fibrillation upon arrival at the emergency department. Subsequently, ECMO therapy (extracorporeal CPR [ECPR]) was rapidly initiated to provide early hemodynamic support and reduce organ ischemia and dysfunction.^[11]^ Approximately 1 hour after ECMO initiation, the patient regained a spontaneous cardiac rhythm, significantly connected to the improved cardiac perfusion provided by ECMO. Studies show that ECPR can increase patient survival rates from 9.9% to 24% for traditional CPR^[12]^ and increase the rate of good neurological outcomes (cerebral performance category, 1–2 or glasgow outcome scale, 4–5) from 5% to 6% for traditional CPR to 18%.^[13]^ Therefore, the good neurological prognosis in this patient was attributed to high-quality CPR and timely ECPR intervention.

Current research highlights the importance of hypothermia therapy in minimizing secondary injuries.^[14]^ Target temperature management is the only proven intervention to effectively improve neurological outcomes in patients with CA. The earlier it is initiated, the greater the benefit to the patient.^[15]^ In this patient, head cooling with an ice cap was initiated during emergency CPR, followed by body surface cooling with an ice blanket combined with an ECMO heater and CRRT upon admission to maintain temperature control. A hibernation mixture was administered to prevent shivering and achieve a target temperature of 35 °C within 1 hour as used to be. Studies show that cooling rates have varying effects on neurological outcomes. A clinical meta-analysis^[16]^ concluded that rapid cooling to a target temperature of 34.8 °C within 3.5 hours post-resuscitation improves neurological outcomes. In this case, rapid cooling contributed to favorable conditions for a good neurological prognosis. Current cooling techniques include intravascular, surface, and body cavity cooling. Intravascular and surface cooling are the most widely used in clinical settings. Owing to the advantages of intravascular cooling in terms of rapid and precise temperature control, it is the preferred method if the conditions allow, with secondary options when necessary.^[15]^ However, a 2013 randomized controlled clinical trial revealed that prehospital intravenous infusion of ice-cold saline to induce hypothermia did not improve survival or neurological outcomes and did not increase the risk of pulmonary edema or recurrent CA^[17]^; therefore, this method is not recommended. In addition, a pig CA model^[18]^ demonstrates that CRRT can induce hypothermia more effectively than traditional surface cooling, offering greater protection for PACS. CRRT allows for temperature control of fluids administered via heated catheters, qualifying it as a form of intravascular cooling. This method avoids the risk of pulmonary edema or volume overload caused by the infusion of ice-cold saline, making it more advantageous for managing patient fluid balance. In addition, hypothermia reduces oxygen consumption, lowers CO demand, and decreases the use of vasopressor drugs, which aids hemodynamic stabilization.^[14]^ The patient was weaned off vasopressor drugs ≈48 hours after admission, with stable hemodynamics, creating an optimal environment for subsequent treatment. Effective temperature control minimizes cerebral metabolic rate, controls ICP, and reduces the production of oxygen-free radicals in the brain. Rewarming began after 5 days, with stable temperature control and no occurrence of high fever, and ICP remained at normal levels after rewarming.

The patient developed acute kidney injury and metabolic acidosis, prompting immediate initiation of CRRT after admission. CRRT maintains internal environmental stability, facilitates fluid balance, and eliminates some inflammatory mediators. CA causes hypoxia, anaerobic metabolism, intracellular acidosis, ATP depletion, ion pump failure, intracellular Ca++ accumulation, and cell edema.^[19]^ With ROSC, these injuries are exacerbated,^[20]^ and ischemia-reperfusion injury triggers an inflammatory cascade reaction, causing a systemic inflammatory response or a sepsis-like syndrome.^[21]^ High levels of inflammatory markers, including procalcitonin, C-reactive protein, IL-6, and IL-10, are associated with poor outcomes following ROSC.^[21]^ In addition, IL-1β, IL-6, IL-10, and tumor necrosis factor-α levels are related to postcardiac arrest syndrome severity.^[22]^ Among these markers, IL-6 has been extensively studied. IL-6 is a cytokine released in response to acute inflammation stimuli or tissue injury, causing vasodilation and endothelial leakage, leading to circulatory instability. Elevated serum IL-6 levels are associated with cardiogenic shock^[23,24]^and poor outcomes following CA.^[25–27]^ Therefore, multiple studies^[28,29]^ aimed to improve patient hemodynamics and prognosis by reducing IL-6 levels. The oXiris filter (Baxter, Meyzieu, France), a device that integrates cytokine and endotoxin removal with renal replacement and antithrombotic functions,^[30]^ is currently used primarily for patients with sepsis. Studies^[31–33]^ show that oXiris can improve the hemodynamic status of patients with sepsis by removing inflammatory mediators, such as tumor necrosis factor-α, IL-6, IL-8, and interferon-γ, ultimately improving patient outcomes. Following oXiris-CRRT, the patients exhibited reduced IL-6 levels, indicating effective control of the inflammatory response. Unfortunately, IL-6 levels were not measured in this patient prior to CRRT. However, the hemodynamics of the patient stabilized rapidly, and vasopressor drugs were discontinued 2 days later. This facilitated early fluid balance management and reduced the risk of cerebral edema. On the same day, CRRT was used for volume management, helping to maintain a negative fluid balance. Consequently, the optic nerve sheath diameter of the patient remained within the normal range, middle cerebral artery blood flow improved, and neuron-specific enolase levels decreased, indicating the absence of severe intracranial hypertension. In addition, cerebral ischemia and hypoxia were rapidly resolved. This contributed significantly to a good neurological prognosis in this patient.

Therefore, for patients undergoing CPR, we need a bundle that includes chest compressions, ECPR if necessary, temperature management, control of brain edema, clearance of inflammatory factors, and so on.

Prolonged CPR often results in severe neurological dysfunction, with cases of minimal neurological impairment being rare in clinical practice. We present the case of a patient who underwent 152 minutes of CPR, regained consciousness within 1 week, and was discharged with full recovery in 2 weeks. However, the pathophysiological mechanisms underlying this recovery process are not well understood, consequently requiring further investigation. This case highlights the potential benefits of the oXiris filter for CRRT in patients who undergo ECPR, suggesting that clinicians should consider using it.

## 4. Conclusion

ECPR is an effective and advanced form of extracorporeal life-support technique used in the treatment of patients with CA. Mechanical CPR devices ensure the delivery of high-quality CPR. CRRT contributes significantly to patient management by regulating temperature, controlling fluid volume, and removing inflammatory mediators, thereby facilitating cerebral recovery.

However, the generalizability of this case is limited due to its singular nature, and further studies involving larger cohorts are necessary to validate these findings. In addition, the potential risks and long-term effects of prolonged CPR, especially in terms of multiorgan failure and complications, warrant further investigation.

## Acknowledgments

The authors would like to thank Editage (www.editage.cn) for English language editing.

## Author contributions

**Conceptualization:** Ya-Dong Wang, Xu-Dong Han.

**Formal analysis:** Ya-Dong Wang, Jin-Feng Lin.

**Project administration:** Ya-Dong Wang, Jin-Feng Lin.

**Resources:** Ya-Dong Wang, Su-Yan Zhang.

**Supervision:** Ya-Dong Wang, Zhi-Long Cao.

**Writing – original draft:** Ya-Dong Wang, Su-Yan Zhang.

**Methodology:** Zhi-Long Cao.

**Investigation:** Su-Yan Zhang, Xu-Dong Han.

**Software:** Su-Yan Zhang, Xu-Dong Han.

**Visualization:** Su-Yan Zhang.

**Writing – review & editing:** Su-Yan Zhang, Xu-Dong Han.

**Data curation:** Xu-Dong Han.
